# ProTstab – predictor for cellular protein stability

**DOI:** 10.1186/s12864-019-6138-7

**Published:** 2019-11-04

**Authors:** Yang Yang, Xuesong Ding, Guanchen Zhu, Abhishek Niroula, Qiang Lv, Mauno Vihinen

**Affiliations:** 1 School of Computer Science and Technology, Soochow University, Suzhou, China; 2 Department of Experimental Medical Science, BMC B13, Lund University, Lund, Sweden; 3 Provincial Key Laboratory for Computer Information Processing Technology, Soochow University, Suzhou, China

**Keywords:** Protein stability, Prediction, Machine learning, Proteome properties

## Abstract

**Background:**

Stability is one of the most fundamental intrinsic characteristics of proteins and can be determined with various methods. Characterization of protein properties does not keep pace with increase in new sequence data and therefore even basic properties are not known for far majority of identified proteins. There have been some attempts to develop predictors for protein stabilities; however, they have suffered from small numbers of known examples.

**Results:**

We took benefit of results from a recently developed cellular stability method, which is based on limited proteolysis and mass spectrometry, and developed a machine learning method using gradient boosting of regression trees. ProTstab method has high performance and is well suited for large scale prediction of protein stabilities.

**Conclusions:**

The Pearson’s correlation coefficient was 0.793 in 10-fold cross validation and 0.763 in independent blind test. The corresponding values for mean absolute error are 0.024 and 0.036, respectively. Comparison with a previously published method indicated ProTstab to have superior performance. We used the method to predict stabilities of all the remaining proteins in the entire human proteome and then correlated the predicted stabilities to protein chain lengths of isoforms and to localizations of proteins.

## Background

Stability is one of the most fundamental properties of molecules. Protein stabilities have been determined with several experimental methods including calorimetric, denaturation and optical spectroscopy approaches. The number of known proteins and their sequences is growing rapidly, but the characterization of their properties is lagging far behind. Stability is of great interest because it is related to most studies and applications of proteins e.g. in medicine and biotechnology.

The available experimental protein stabilities have been obtained in vitro, but in vivo stabilities can be different due to many cellular effects. Some methods have been developed for the prediction of protein stability, especially for melting temperature, T_m_. These tools are based on different principles, including amino acid sequences [1, 2], protein chain lengths [3, 4], physicochemical features [5], living temperature of organism and salt bridges [6], temperature-dependent statistical potentials [7, 8], and descriptors of protein surface [9], and reviewed in [10]. Numerous additional factors have been shown to have correlation with protein stability, including flexibility [11, 12], hydropathy [13], hydrogen bonding [14], packing [15] and others.

Some of the predictions are rather simple to calculate, such as lengths of protein sequences. More advanced machine learning (ML) methods have utilized decision trees and neural networks (NN) [5], and NNs and adaptive network-fuzzy inference system (ANFIS) [1].

Substantially larger number of prediction methods forecast effects of single amino acid substitutions on protein stability. Energy function-based methods use either physical energy function from ab initio quantum mechanics (QM) calculations, empirical energy function or force field, or statistical energy function. ML-based methods form the other major group. These tools are used to predict the sign of ΔΔG (stabilizing/destabilizing), the value of ΔΔG, or both. A wide array of algorithms have been used, including gradient boosting [16], neural networks [17, 18], random forests [19–21], support vector machines [21–25], and a metapredictor [26]. All these tools have been trained with data from the same source, ProTherm database [27]. Performances of these methods vary widely [28, 29]. Recently, we noticed a number of problems and issues with ProTherm and therefore cleaned and pruned the data before developing a novel predictor [21].

The overall protein stability prediction methods have suffered from limited amounts of available experimental data. Therefore, many of the existing tools are based on very small numbers of known cases, which negatively affects the performance of methods since stability is a complex property and several features contribute to it. Small sample sizes do not allow identification of all dependencies.

The situation has changed recently when a cellular stability method based on limited proteolysis and mass spectrometry (LiP-MS) was introduced and applied to cell-wide analysis of protein stability in four organisms, namely *Escherichia coli*, *Homo sapiens*, *Saccharomyces cerevisiae* and *Thermus thermophilus* [30]. This dataset for altogether 3520 proteins was used to train a gradient boosting-based ML method called ProTstab. The method has good performance and is suited for large scale prediction since it is very fast. We used ProTstab to predict stabilities of the remaining proteins in human proteome and their all sequence isoforms and correlated them to sensitivity of these proteins for harmful variants and to subcellular localization of proteins and isoform lengths.

## Results

The availability of novel high-throughput dataset [30] facilitated the development of a reliable predictor for cellular protein stability. Gradient boosting-based method was trained, tested and applied to prediction of various cases.

### Method training and development

We used RFECV (Recursive feature elimination) algorithm since it has been successful in previous bioinformatics applications including the development of support vector machine (SVM) and random forest (RF) classification and regression predictors [31, 32].

We trained seven regression predictors with the top 50, 100, 200, 300, 500, 1000 features or with all the 2077 features (Table 1). To avoid problems with excessive number of features that can cause overfitting and other problems, we chose the best predictor with the smallest number of features. The best performance was obtained with 100 features, and we call the tool as ProTstab. The features and their importance scores are given in Additional file 1: Table S1. Overall, the importance scores are very small, indicating small impact of individual features, however together they yield rather good performance. Group 5 frequency has the highest impact. Amino acids have been classified to six categories based on their properties, group 5 contains residues N, Q and S. The other informative features represent numerous types of characteristics.

**Table 1:** Performance of prediction methods on 10-fold cross validation and blind test

	Performance with top importance features						
Measure	50	100	200	300	500	1000	2077
PCC	0.790	0.793	0.790	0.786	0.779	0.772	0.767
RMSE	0.165	0.164	0.165	0.166	0.169	0.171	0.173
R^2^	30.5	47.8	28.2	35.7	39.2	27.7	32.4
MSE	0.030	0.024	0.030	0.028	0.026	0.029	0.026
MAE	0.133	0.125	0.141	0.133	0.134	0.133	0.135
Blind test							
Blind PCC	0.702	0.736	0.735	0.740	0.756	0.755	0.758
Blind RMSE	0.197	0.189	0.189	0.187	0.183	0.184	0.183
Blind R^2^	−10.9	−8.5	−12.2	−5.1	−1.1	−5.20	−6.7
Blind MSE	0.039	0.036	0.036	0.035	0.033	0.034	0.033
Blind MAE	0.160	0.146	0.145	0.145	0.142	0.142	0.143

Five measures were used to chart the full performance of the predictors. We tested the methods both in 10-fold cross validation (CV) as well as with a blind test set separated in the beginning and not used during training (Table 1). ProTstab has the highest PCC and the lowest RMSE both in the 10-fold CV and in blind test, 0.793 and 0.763, and 0.164 and 0.189, respectively. R^2^ indicates the goodness of fit of a model on how well the regression predictions approximate the real data points. Value of 1 indicates perfect fit to the data. In the CV R^2^ indicates that about half (47.8%) of the data is explained by the model. The corresponding number for the blind set is 8.5%.

### Comparison to other methods

We wanted to compare the performance of ProTstab to published tools, presented in the Introduction. However, this was possible only for the method of Ku et al. [2] since the other methods were not available as service or for download. The tool of Ku and coworkers is somewhat different, as the statistical method classifies proteins into three melting point categories (T_m_ > 65, T_m_ < 55, or 55 < T_m_ < 65). We submitted proteins in our blind test dataset to the web service [33]. For these proteins, the classification accuracy of ProTstab is 0.60 (180 correct out of 300) and for the Ku et al. predictor it is significantly lower, 0.38 (114 correct out of 300). The low accuracy likely reflects the small size of the training set, only 35 proteins.

A recent publication analyzed and discussed the relations between T_m_ and a series of factors that are expected to influence protein stability [10]. These factors were then combined to build an improved prediction method. It has a very good performance and very low published RMSE value on their test data but may suffer from the very small training dataset of only 45 proteins. This method could not be compared as it is not publicly available. We used their 45 proteins for blind testing ProTstab, gaining low performance (PCC 0.40, RMSE = 0.26 after normalization).

**Fig. 1:**
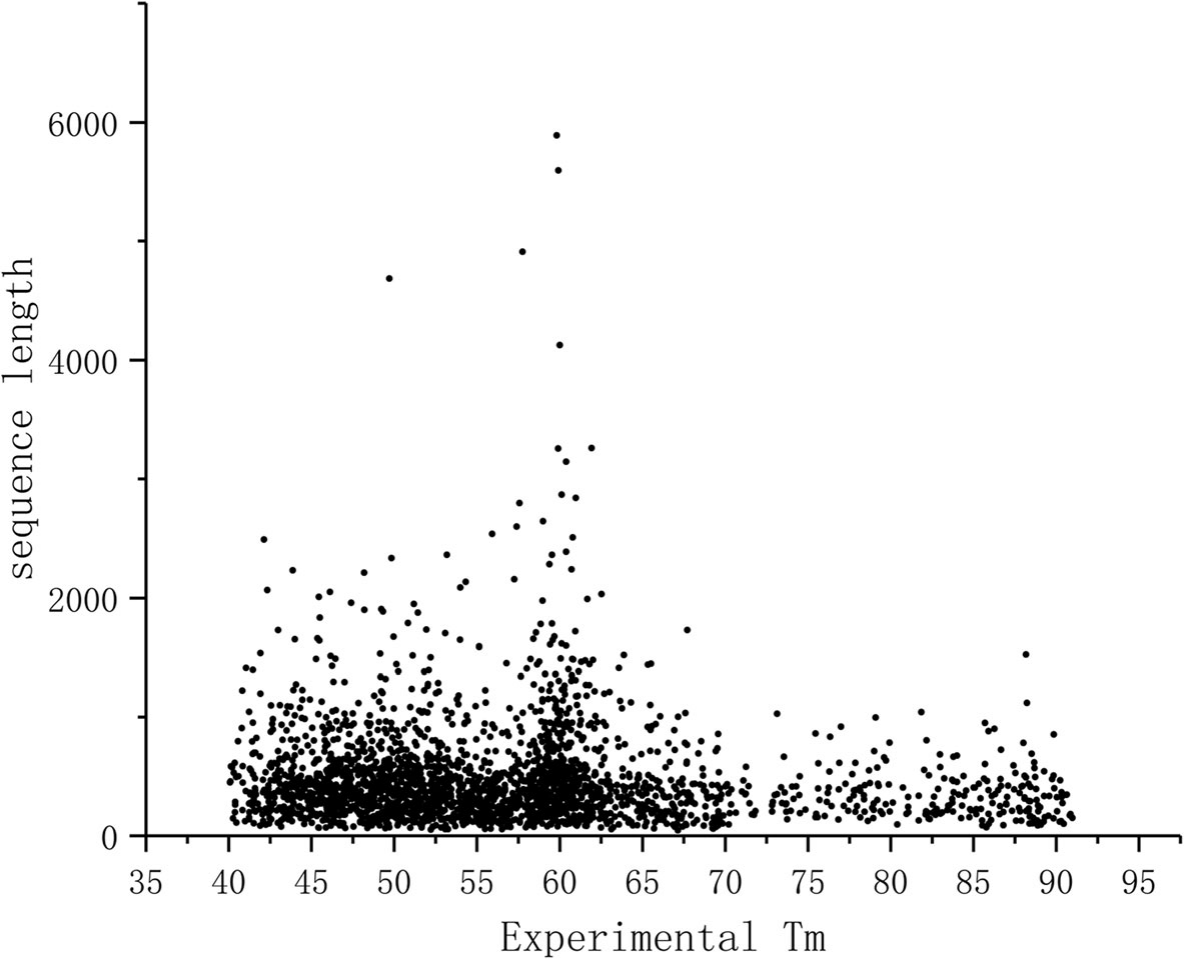
Correlation of protein length and Tm for the experimentally defined training dataset

Previously, sequence length has been considered as a strong predictor of stability [3, 4]. Figure 1 clearly shows that there is no correlation between protein chain length and the experimental T_m_ values (PCC = − 0.237) and thus this feature cannot be used for predictions. Sequence length was not among the features used for training ProTstab. On the ranked list of features it is on position 1903 out of 2077 i.e. towards the end of least significant features.

### Distribution of stabilities in isoforms

Numerous proteins appear in several isoforms due to alternative translation initiation, alternative mRNA splicing, proteolysis or other post translational modifications. Analyses of N-terminal [34] and entire proteomes [35] showed that isoforms often have different cellular stabilities (turnover rates). The turnover has strong correlation with thermal stability.

**Fig. 2:**
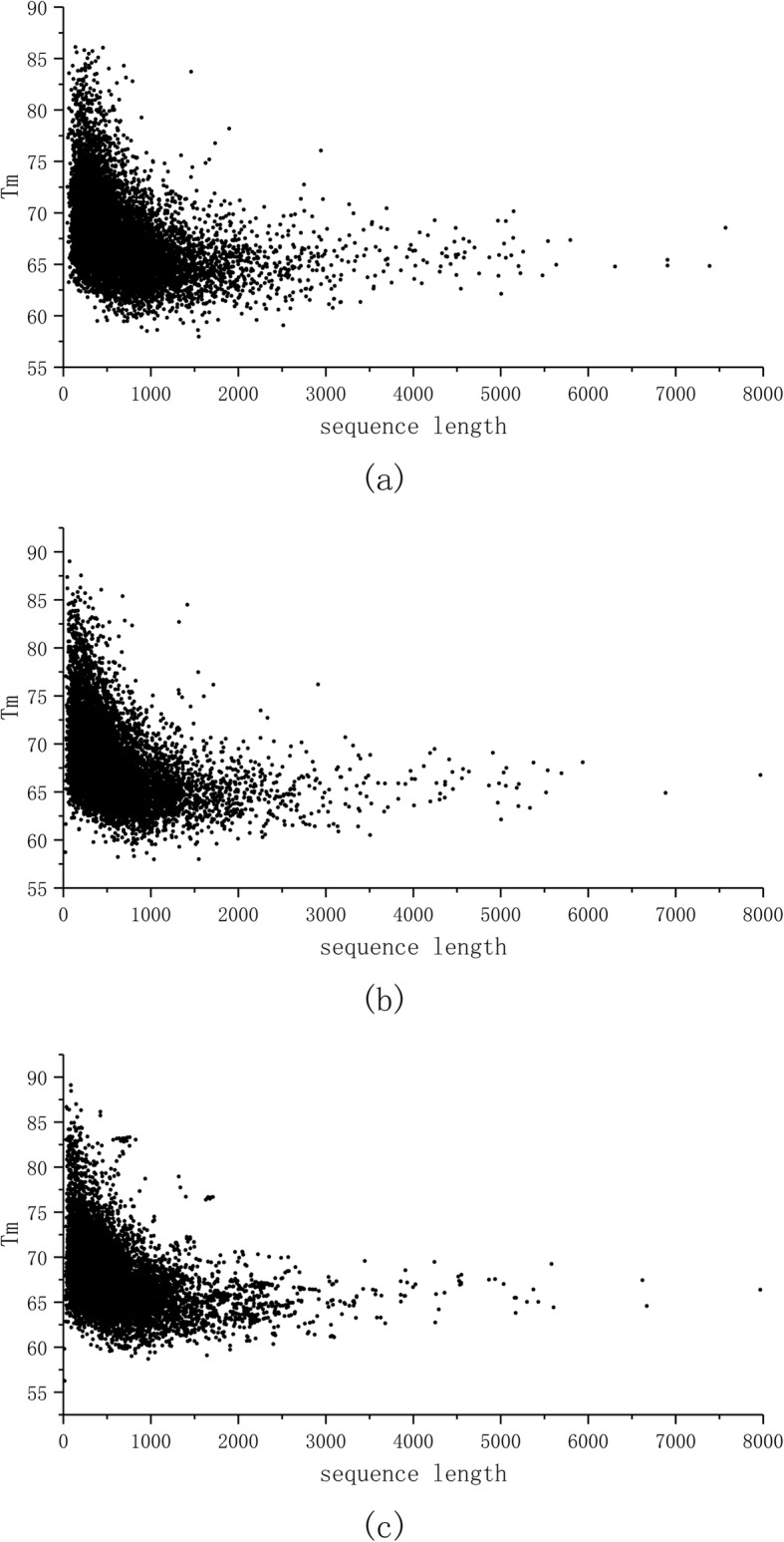
Differences in predicted stabilities of isoforms vs chain length. Top, the longest isoform, middle, second longest isoform; bottom: other isoforms. Data are only for proteins with at least two isoforms. The graphs show melting temperature (T_m_) vs protein sequence length

We predicted stabilities for all isoforms in all the human proteins to study whether isoform length correlates with stability. In Fig. 2 a is shown the distribution of the predicted stabilities for the longest isoforms of human proteins and they do not differ from those for the second longest isoform (Fig. 2b) or for even shorter isoforms (Fig. 2c). Only proteins that had at least two isoforms of different lengths were included to the analysis. The PCC values is − 0.288 for data in Fig. 2a, − 0.298 in Fig. 2b, and − 0.186 in Fig. 2c, all indicating lack of significant correlation between protein stabilities and isoform chain lengths.

Collectively Figs. 1 and 2 showed that protein chain length is not correlated to cellular stability whether for different forms of the same protein or for different proteins. Chain length was one of the features describing our proteins, however was not among the selected important features, actually being among the least significant features.

### Stability and overall sensitivity of proteins for substitutions

Proteins present widely different vulnerabilities for amino acid substitutions. We have previously investigated the sensitivity of nine groups of proteins for all 19 possible amino acid substitutions in all positions [36]. The sensitivities of the proteins were obtained by predicting with a highly reliable variant pathogenicity/tolerance tool PON-P2 [37]. The studied groups were for actionable, cancer, cardiologic, developmental, epilepsy, neurodegenerative, and primary immunodeficiency diseases, as well as for housekeeping and non-disease non-housekeeping proteins [36].

The results for 929 unique proteins in the 9 groups (some of the proteins belong to more than one group) indicated that the sensitivity, i.e. the ratio of harmful variants, varies greatly for proteins that tolerate almost all possible single amino acid substitutions to those in which only a very small number of variants are considered to be benign. However, a number of tendencies were seen between the groups.

**Fig. 3:**
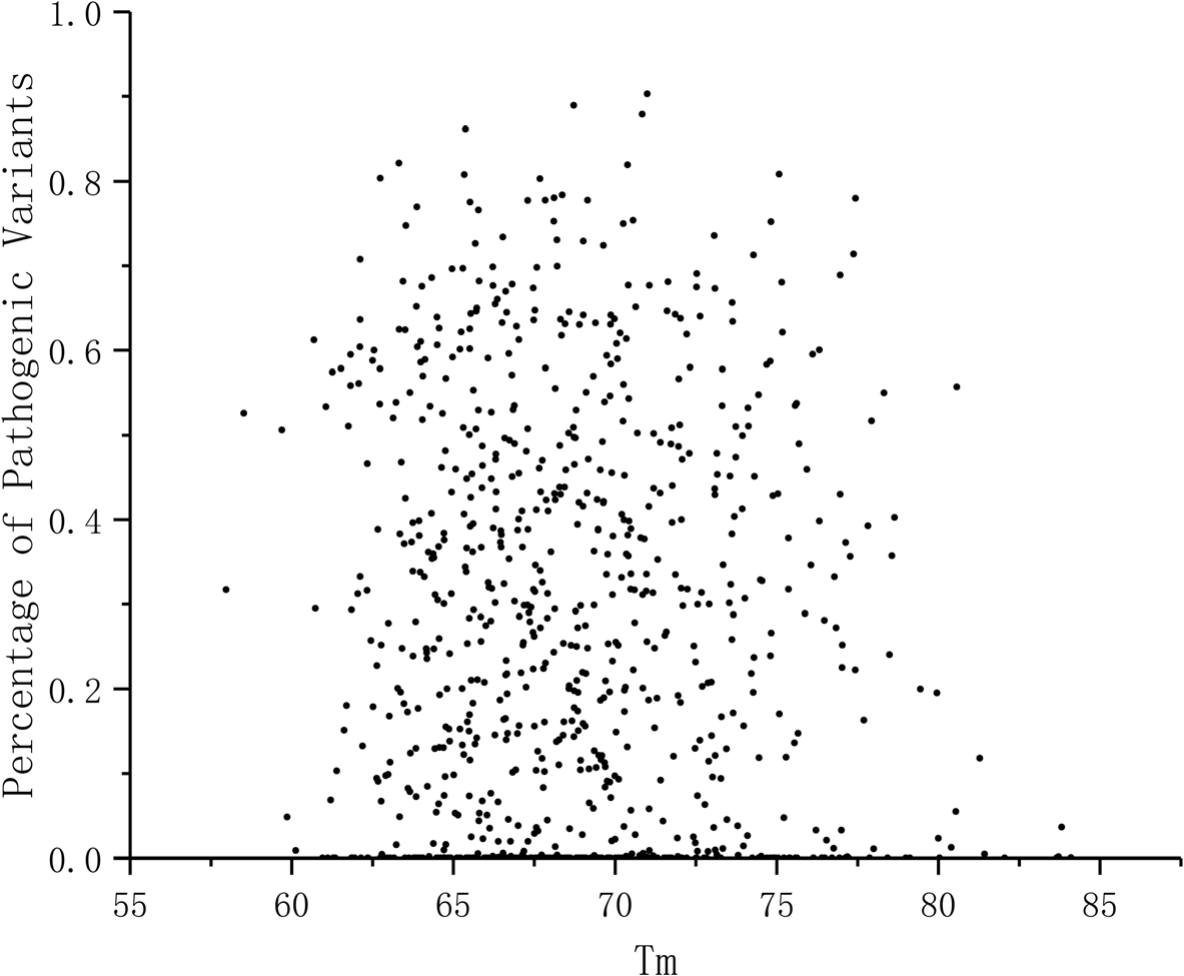
Analysis of the relationship of T_m_ to predicted sensitivity for harmful variants

We correlated protein T_m_ values to the predicted percentage of pathogenicity of variants and plotted Fig. 3. There is no significant correlation (PCC = -0.286), instead the stability values show very random distribution. Thus, although effects on protein stability are among the most common effects for disease-causing amino acid substitutions [38, 39], the sensitivity for these variants does not correlate with T_m_.

### Protein localization and stability

Proteins are localized to various compartments within cells or secreted outside of them. The environments within the compartments are widely different, therefore one might expect it to be reflected to the stabilities of the included proteins. To address this, we obtained the most common localizations of proteins from Human Protein Atlas [40], where there were data for 19,327 proteins.

Since several proteins can localize to several compartments, we concentrated on the major compartments for every protein. The results are in Additional file 1: Figure S1. Totally 20 compartments contained at least 100 proteins and were included to the analysis. The T_m_ distributions are practically identical in all the tested compartments, thus protein stabilities are similar throughout the cells irrespective of the organelles.

### Stabilities in human proteome

In Additional file 1: Figure S2 is shown the overall distribution for predicted human protein stabilities. Since ProTstab was found to have good performance, we used it to predict stabilities of all human proteins and isoforms not included to the training set. These data are available at our website at [41]. Experimental data were available for 1009 human proteins in our training set. The predictions contain stabilities for 32,117 proteins and isoforms.

## Discussion

Altogether seven predictors were trained with different numbers of features. ProTstab has the best scores for all the measures for CV data indicating that the top 100 features optimally capture the property space (Table 1). Similar result was obtained with the blind dataset, although some individual scores were slightly better for some other feature combinations.

Three out of the five quality measures used indicate errors in predictions (RMSE, MSE and MAE). The smaller the scores, the better the method is. All these values indicate ProTstab to be reliable. The scores are better for all the tested predictors on the CV data. In conclusion, the performance is good and the method can be used for various applications. The tool can be used to predict stabilities for proteins from any organism and of any length as it has been trained to generalize from proteins with different origin and properties. However, we anticipate that very short proteins or polypeptides, shorter than ~ 40 amino acids, would be predicted with lower accuracy, because these molecules are usually not well ordered, whereas the features are for compact molecules with sigmoidal denaturation patterns.

Most of the previous methods suffer from small datasets. Only 22 proteins were used for the development of Volsurf [9]. The sequence length methods are based on 65 proteins [3, 4], and the growth environment method on 127 proteins [6], and those using temperature-dependent statistical potentials on 45 [8] and 166 proteins [7]. The amino acid sequence-based method was trained on 230 proteins [1]. These numbers are very small considering the difficulty of the task. The only tool with a larger dataset of 2057 proteins [5] does not provide any information about the included proteins, their origin, stability or other details. This method is not available, either. Thus, it was impossible to compare with these methods. ProTstab was trained with significantly larger dataset of 3520 proteins than the others.

ProTstab could be compared only to one previously published method, and it showed superior performance. We could extent the comparison to two additional methods which are based on sequence length [3, 4]. Our results in Fig. 1 indicate that protein chain length does not correlate with the stability. This is evident also from the ranking of features, the chain length is on position 1903 among the 2077 features tested, i.e. it is among features with least significance. As a further test for the relationship of polypeptide chain length and stability we predicted the stabilities of all alternative protein isoforms (Fig. 2). The distributions are identical in all cases, thus also this analysis indicated missing correlation between chain length and stability.

Proteins are known to show different vulnerability for amino acid substitutions. PON-P2 is a highly reliable predictor of variant pathogenicity. T_m_ values and protein sensitivity do not show correlation (Fig. 3), which was not even expected as the sensitivity is a sum of very large number of factors. To further test the properties of proteins and their relation to stability we investigated 20 subcellular localizations of human proteins for which there were at least 100 proteins in the dataset (Additional file 1:Figure S1). The distributions are very similar for all the tested compartments.

Finally, we shared the predicted stabilities for all the isoforms in the human proteome and made the dataset publicly available. We believe that ProTstab will be a valuable tool for estimating protein cellular stability in various organisms. Stability is an important property and affects many experimental studies such as protein production, purification and characterization and can be modified with protein engineering with the help of the developed tool.

## Conclusions

Knowledge of protein stabilities has numerous applications in experimental design, protein structural studies, expression, purification, medical applications, biotechnology etc. Although some tools have been presented for the prediction of protein cellular stability they have been based on very small datasets and thus had poor performance. We utilized a novel large-scale dataset and trained an ML predictor that has good performance. The method can be used for predictions of all kinds of proteins irrespective of origin allowing also designed ones to be predicted. The method was used to predict all the human proteins and their length isoforms. The results were correlated to the protein chain length, sensitivity of proteins for substitutions, and protein subcellular localization. No major correlations were seen in these studies. ProTstab can be used for predictions of proteins from any source or size, possibly excluding short polypeptides that do not have well defined structures.

## Methods

### Dataset

We used a dataset of 3520 proteins, 729 from *E. coli*, 709 from *S. cerevisiae*, 1073 from *T. thermophilus*, and 1009 from human [30]. Three hundred proteins (*E. coli* 60, *S. cerevisiae* 60, *T. thermophilus* 90, human 90) were extracted and randomly partitioned as a blind test dataset. The remaining 3220 proteins were used for method development. Sequences for the proteins were obtained from UniProtKB [42]. The dataset is available from VariBench [43] at [44].

### Features

A large number of features were collected to describe characteristics of proteins. These include physicochemical, structural, and composition features that describe properties of entire proteins. The features were generated with three services. PROFEAT [45] calculates structural and physicochemical features from amino acid sequences. PROTEIN RECON [46] provides protein charge density-based electronic properties based on atomic charge density fragments computed from ab initio wave functions. The method is based on the quantum theory of atoms in molecules (QTAIM) [47]. ProtDCal [48] was used to generate sequence-based descriptors. On top of these features we included protein chain length, molecular weight, isoelectric point, CHNSO (carbon, hydrogen, nitrogen, sulphur, oxygen) counts for element types and their frequencies, 6 amino acid group counts and frequencies, count and frequencies of negatively charged, positively charged, hydrophilic and hydrophobic residues, as well as dipeptide counts. After removal of redundant ones we had altogether 2077 features, of which 1437 were from ProFEAT, 140 from PROTEIN RECON, and 19 from ProtDCal. We used sequence-based features since three dimensional structures were not widely available for the proteins for which there was stability information. Further, we wanted to develop a generic predictor and therefore structure-based features were not included.

### Regression algorithm

Gradient boosting machine learning algorithm [49] was trained for regression to predict T_m_ values. Gradient boosting of regression trees (GBRT) is a general ML technique for classification and regression. The algorithm is highly resistant to overfitting. GBRT combines weak regression models iteratively into a single strong model to minimize the mean squared error (MSE) of prediction value, according to the empirical risk minimization (ERM) principle. It utilizes the residuals between prediction values and actual values at each stage of iteration to improve the original weak model, i.e. the original regression tree model.

We used Scikit-learn toolkit [50] to implement the GBRT training and testing. Hyper parameters were tuned with a grid-based search. The maximum depth (max_depth) and the minimum required number of samples at a leaf (min_samples_leaf) were set as 3 for each tree, and the total number of regression trees (n_estimators) was set to 3000.

### Feature selection

Previously, numerous factors have been presented to correlate with protein stability. We collected a very large set of characteristics and used all of them as training features for GBRT algorithm. As too many features may lead to problems including lowered prediction performance, longer training times and overfitting, we performed a feature selection based on feature importance ranking.

In GBRT, the rank (i.e. depth) of a feature as a decision node in a tree can be utilized to assess the relative importance of the feature in respect to the predictability of the target variable. Features used at the top of the tree contribute to a larger fraction of input samples and have thus higher relative rank. The expected fraction of the samples each feature contributes to was used as an estimate of the relative importance of the feature [50].

We used recursive feature elimination with CV in the GBRT algorithm and implemented with the Python package Scikit-learn toolkit. By recursively eliminating features ranked with low importance and using cross validated selection to optimize the features selected for regression, we got a list of sieved features and used them to train GBRT predictors.

### Performance assessment

We used totally five measures to describe and estimate the method performance in regression.

Pearson correlation coefficient (PCC) is defined as the covariance of the two variables (*X* and *Y*, in our case experimental and predicted values) divided by the product of their standard deviations. It provides a correlation between the two variables, as follows

$$ \mathrm{PCC}=\frac{N\sum XY-\sum X\sum Y}{\sqrt{N\sum {X}^2-{\left(\sum X\right)}^2}\sqrt{N\sum {Y}^{2-}{\left(\sum Y\right)}^2}}, $$

where *N* is the number of data items.

The root mean square error (RMSE) measures the differences between predicted and experimental values. The RMSE represents the sample standard deviation of the differences between predicted and observed values:

$$ \mathrm{RMSE}=\sqrt{\frac{\sum_{i=1}^N{\left({Y}_i-{X}_i\right)}^2}{N}}. $$

Mean absolute error (MAE) measures the difference between predictions and real values

$$ MAE=\frac{\sum_{i=1}^N\mid {Y}_i-{X}_i\mid }{N}. $$

Mean squared error (MSE) measures the average of the squares of errors as follows

$$ MSE=\frac{1}{N}{\sum}_{i=1}^N{\left({Y}_i-{X}_i\right)}^2, $$

where Y_i_ is a vector for predictions and *X*_*i*_ is a vector for observations. *N* is the total number of predictions.

The *R*^*2*^ provides the percentage of variation explained by the model with the approach of least squares. In regression, *R*^*2*^ estimates how close the data are to the fitted regression line. The better the regression model, the closer the value is to 1. The most general definition of the *R*^*2*^ is

$$ {R}^2\equiv 1-\frac{SS_{res}}{SS_{tot}}=1-\frac{\sum_i{\left({X}_i-Y\right)}^2}{\sum_i{\left({X}_i-\overline{X}\right)}^2}, $$

where *ss*_*res*_ is sum of squares of residuals and *ss*_*tot*_ is total sum of squares.

### Correlation of predictions to protein properties

The predictions with ProTstab were correlated with data for various aspects related to proteins. Information for protein isoforms were obtained from UniProt database. Protein subcellular localizations were retrieved from Human Protein Atlas (HPA) [40]. PCC was used to reveal the significance of the observations.

### Implementation of web service

The web service of ProTstab was implemented using a free and open source framework Django based on Python language [51]. Users submit a sequence in FASTA format along with a protein name. The prediction result will then be sent back by email after calculation. There is also a batch submission available for simultaneous submission of several protein sequences. The web service is freely available at [52]. There are also pre-calculated results for all human proteins and their isoforms.

## Supplementary information

**Additional file 1: Table S1.** Numbers of proteins in membrane subcellular localizations. **Table S2.** Performance of subcellular localization predictors on MP1289 restricted to one subcellular localization per protein. **Table S3.** Performance of subcellular localization predictors on single and multi pass membrane proteins. **Figure S1.** Distributions of stability values within the most populated subcellular localizations. **Figure S2.** Distribution of the predicted stabilities of human proteins.

## Data Availability

ProTstab predictor is freely available at http://structure.bmc.lu.se/ProTstab. Data used for training and testing are available in the VariBench database at http://structure.bmc.lu.se/VariBench/protein_stability. Predictions for all human proteins are available at http://structure.bmc.lu.se/ProTstab/HumanProteomeStabilities.

## Abbreviations

ANFIS: Adaptive network-fuzzy inference system
CHNSO: Carbon, hydrogen, nitrogen, sulphur, oxygen
CV: Cross validation
ERM: Empirical risk minimization
GBRT: Gradient boosting of regression trees
HPA: Human Protein Atlas
LiP-MS: Limited proteolysis and mass spectrometry
MAE: Mean absolute error
ML: Machine learning
MSE: Mean squared error
NN: Neural network
PCC: Pearson correlation coefficient
QM: Quantum mechanics
QTAIM: Quantum theory of atoms in molecules
RF: Random forest
RFECV: Recursive feature elimination
RMSE: Root mean square error
SVM: Support vector machine
