# Supplementary material for: ProTstab – predictor for cellular protein stability
Source: BMC Genomics. 2019 Nov 4;20:804. doi: 10.1186/s12864-019-6138-7 (PMC6830000; doi:10.1186/s12864-019-6138-7)
Supplement: Supplementary file 1 — Additional file 1: Table S1. Numbers of proteins in membrane subcellular localizations. Table S2. Performance of subcellular localization predictors on MP1289 restricted to one subcellular localization per protein. Table S3. Performance of subcellular localization predictors on single and multi pass membrane proteins. Figure S1. Distributions of stability values within the most populated subcellular localizations. Figure S2. Distribution of the predicted stabilities of human proteins. [file 12864_2019_6138_MOESM1_ESM.docx]

**ProTstab – Predictor for cellular protein stability**

**Supplementary material**

Yang Yang^1,2,3^, Xuesong Ding^1^, Guanchen Zhu^1^, Abhishek Niroula^2^, Qiang Lv^1^ and Mauno Vihinen^2,*^

^1^ School of Computer Science and Technology, Soochow University, China

^2^ Department of Experimental Medical Science, BMC B13, Lund University, Lund, Sweden

^3^ Provincial Key Laboratory for Computer Information Processing Technology, Soochow University, China

Supplementary Table 1. Numbers of proteins in membrane subcellular localizations.

| Localization | MP1289 | | MP508 | |
| --- | --- | --- | --- | --- |
|  | No | % | No | % |
| Plasma membrane | 378 | 29.3 | 182 | 35.8 |
| Nuclear membrane | 84 | 6.5 | 32 | 6.3 |
| Cell Junctions | 80 | 6.2 | 37 | 7.3 |
| MLPs | 747 | 58 | 257 | 50.6 |

Supplementary Table 2. Performance of subcellular localization predictors on MP1289 restricted to one subcellular localization per protein.

| Method | BUSCA | CELLO | DeepLoc1.0 | LocTree3 | MultiLoc2 | SubCons | Wolf PSORT |
| --- | --- | --- | --- | --- | --- | --- | --- |
| TP | 149 | 166 | 253 | 183 | 58 | 124 | 263 |
| FP | 8 | 7 | 1 | 0 | 5 | 5 | 19 |
| TN | 403 | 486 | 387 | 537 | 496 | 301 | 523 |
| FN | 279 | 344 | 288 | 358 | 408 | 215 | 279 |
| Sensitivity | 0.35 | 0.33 | 0.47 | 0.34 | 0.12 | 0.37 | 0.49 |
| Specificity | 0.98 | 0.99 | 1.00 | 1.00 | 0.99 | 0.98 | 0.96 |
| PPV | 0.95 | 0.96 | 1.00 | 1.00 | 0.92 | 0.96 | 0.93 |
| NPV | 0.59 | 0.59 | 0.57 | 0.60 | 0.55 | 0.58 | 0.65 |
| ACC | 0.66 | 0.65 | 0.69 | 0.67 | 0.57 | 0.66 | 0.73 |
| MCC | 0.42 | 0.41 | 0.51 | 0.45 | 0.23 | 0.44 | 0.51 |
| OPM | 0.35 | 0.34 | 0.42 | 0.37 | 0.24 | 0.36 | 0.43 |

Supplementary Table 3. Performance of subcellular localization predictors on single and multi pass membrane proteins.

|  | BUSCA | | CELLO | | DeepLoc1.0 | | LocTree3 | | MultiLoc2 | | SubCons | | Wolf PSORT | |
| --- | --- | --- | --- | --- | --- | --- | --- | --- | --- | --- | --- | --- | --- | --- |
| Method | 1 TM | >1 TM | 1 TM | >1 TM | 1 TM | >1 TM | 1 TM | >1 TM | 1 TM | >1 TM | 1 TM | >1 TM | 1 TM | >1 TM |
| TP | 1307 | 2459 | 697 | 2404 | 1879 | 2363 | 1242 | 2635 | 325 | 862 | 871 | 1905 | 1442 | 2574 |
| FP | 104 | 111 | 75 | 76 | 115 | 130 | 75 | 111 | 49 | 66 | 47 | 64 | 113 | 146 |
| TN | 1848 | 2370 | 2228 | 2815 | 1920 | 2471 | 2290 | 2876 | 2174 | 2708 | 1683 | 2131 | 2284 | 2878 |
| FN | 633 | 151 | 1669 | 275 | 231 | 196 | 1117 | 364 | 1453 | 2014 | 787 | 492 | 950 | 448 |
| Sensitivity | 0.67 | 0.94 | 0.29 | 0.90 | 0.89 | 0.92 | 0.53 | 0.88 | 0.18 | 0.30 | 0.53 | 0.79 | 0.60 | 0.85 |
| Specificity | 0.95 | 0.96 | 0.97 | 0.97 | 0.94 | 0.95 | 0.97 | 0.96 | 0.98 | 0.98 | 0.97 | 0.97 | 0.95 | 0.95 |
| PPV | 0.93 | 0.96 | 0.90 | 0.97 | 0.94 | 0.95 | 0.94 | 0.96 | 0.87 | 0.93 | 0.95 | 0.97 | 0.93 | 0.95 |
| NPV | 0.74 | 0.94 | 0.57 | 0.91 | 0.89 | 0.93 | 0.67 | 0.89 | 0.60 | 0.57 | 0.68 | 0.81 | 0.71 | 0.87 |
| ACC | 0.81 | 0.95 | 0.63 | 0.94 | 0.92 | 0.94 | 0.75 | 0.92 | 0.62 | 0.63 | 0.75 | 0.88 | 0.78 | 0.90 |
| MCC | 0.65 | 0.90 | 0.35 | 0.88 | 0.83 | 0.87 | 0.55 | 0.84 | 0.27 | 0.37 | 0.56 | 0.77 | 0.59 | 0.81 |
| OPM | 0.55 | 0.85 | 0.30 | 0.82 | 0.77 | 0.82 | 0.46 | 0.78 | 0.27 | 0.32 | 0.47 | 0.69 | 0.50 | 0.74 |








Supplementary Figure 1. Distributions of stability values within the most populated subcellular localizations.





Supplementary Figure 2. Distribution of the predicted stabilities of human proteins.
